# Tracking Dynamics of Superspreading Through Contacts, Exposures, and Transmissions in Edge-Based Network Epidemics

**DOI:** 10.1007/s11538-026-01685-5

**Published:** 2026-06-25

**Authors:** Ari S. Freedman, Bjarke F. Nielsen, Maximilian M. Nguyen, Laurent Hébert-Dufresne, Simon A. Levin

**Affiliations:** 1 Department of Ecology and Evolutionary Biology, Princeton University, Princeton, NJ USA; 2 Department of Plant Biology, University of Vermont, Burlington, VT USA; 3 Vermont Complex Systems Institute, University of Vermont, Burlington, VT USA; 4 High Meadows Environmental Institute, Princeton University, Princeton, NJ USA; 5 Niels Bohr Institute, University of Copenhagen, Copenhagen, Denmark; 6 PandemiX Center, Roskilde University, Roskilde, Denmark; 7 Lewis-Sigler Institute, Princeton University, Princeton, NJ USA; 8 School of Medicine, Emory University, Atlanta, GA USA; 9 Department of Computer Science, University of Vermont, Burlington, VT USA; 10 Complexity Science Hub, Vienna, Austria; 11 Santa Fe Institute, Santa Fe, NM USA

**Keywords:** Superspreading, Networks, Disease modeling, Dispersion, SARS-CoV-2

## Abstract

Infectious disease superspreading caused by heterogeneity in contact behavior has been observed to be an important determinant of epidemic dynamics and size in both empirical and theoretical settings. However, it has also been observed that the importance of this type of superspreading changes throughout an epidemic, generally in a decreasing manner as infections cascade from individuals with many contacts to those with fewer contacts. We provide an exact mathematical formulation of this phenomenon in strongly-immunizing (SIR) epidemics on static contact networks. Building on the edge-based modeling framework, we construct three metrics to track how superspreading changes through the course of an epidemic, respectively measuring infected nodes’ contacts, exposures, and transmissions: (1) the mean degree of infected nodes, (2) the mean number of susceptible neighbors of infected nodes, and (3) the mean number of secondary cases that will be caused by newly infected nodes. We prove results about the behaviors of these metrics, highlighting the fact that their peak times all occur at less than half the time it takes for population-level infection prevalence to peak. This suggests that the importance of superspreading will be low when an epidemic is already near its peak, so contact-based control strategies are best employed as early in an outbreak as possible. We discuss implications for accurately measuring epidemiological parameters from incidence, mobility, contact tracing, and transmission data.

## Introduction

Infectious disease models traditionally assume individuals in a population are well-mixed in their contact patterns, assuming mass-action transmissions where incidence is proportional to both the number of infected individuals and the number of susceptible individuals (Kermack and Mckendrick 1927; Heesterbeek 2005). However, human populations are highly structured in their contact patterns, a phenomenon which models often capture abstractly using contact networks (Klovdahl 1985; Watts and Strogatz 1998; Newman 2002; Eubank et al. 2004; Balcan et al. 2009). Much work has been done on mathematically describing the spread of infectious disease and other spreading processes on networks using a variety of methods, including branching process (Lloyd-Smith et al. 2005), moment closure (Eames and Keeling 2002; Bauch 2002), degree block (Barrat et al. 2013), approximate master equation (Marceau et al. 2010; Hébert-Dufresne et al. 2010; Gleeson 2013), effective degree (Ball and Neal 2008; Lindquist et al. 2011), message passing (Karrer and Newman 2010; Sherborne et al. 2018), generation-based (Noël et al. 2009) and edge-based approaches (Volz 2008; Miller 2011). Particular emphasis has been placed on models with perfect immunity, adapting the original SIR model of Kermack and Mckendrick (1927) to a static contact network with an arbitrary degree distribution. Of these, Volz (2008) achieved the first low-dimensional mathematical description of a SIR epidemic evolving over a network in continuous time, an approach further simplified by Miller (2011) down to a system of two ordinary differential equations.

A motivating force behind this proliferation of network infectious disease models has been the increasingly recognized significance of superspreading in epidemics (Klovdahl 1985; Lloyd-Smith et al. 2005; Brainard et al. 2023), where a small group of individuals are responsible for a majority of transmissions (during the SARS-CoV-2 pandemic, 10% of cases caused as much as 80% of infections Miller et al. 2020; Endo et al. 2020; Lau et al. 2020; Althouse et al. 2020). Superspreading events can be driven by various factors, from heterogeneity in biological factors like viral shedding volume to behavioral factors such as the size of one’s social contact network (Nielsen et al. 2023; Goyal et al. 2021; Althouse et al. 2020). Here, we focus on contact heterogeneity, which has been shown to have important effects on epidemic dynamics (Lloyd-Smith et al. 2005; Volz 2008; Miller et al. 2012), critical outbreak threshold (Andersson 1997; May and Lloyd 2001; Newman 2002; Pastor-Satorras and Vespignani 2002; Miller et al. 2012), final size (Miller 2011; Großßmann et al. 2021; Noël et al. 2009), herd immunity threshold (Britton et al. 2020; Gomes et al. 2022; Oz and Rubinstein 2021), extinction probabilities (Lloyd-Smith et al. 2005; Diekmann et al. 2012), evolutionary potential (Leventhal et al. 2015), and effectiveness of control efforts (Lloyd-Smith et al. 2005; Kiss et al. 2005; Nielsen et al. 2023).

Another previously described phenomenon of epidemic spreading on heterogeneous networks is the propensity for high degree nodes (representing individuals with many contacts) to be more likely to become infected and to do so earlier, as they are the ones most likely to be connected by a random edge (or contact) in the network (Allard et al. 2023; Barthélemy et al. 2005). This is the same logic underlying the “friendship paradox”, that a random friend of an individual will on average have more friends than that individual (Allard et al. 2023). Furthermore, if the infection has long-lasting immunity, then the infection will tend to cascade from high degree nodes to low degree nodes as the high degree nodes become infected first but then also recover and gain immunity first (Barthélemy et al. 2005). Thus, the potential for superspreading in an epidemic may decline over time. Statistical approaches have been developed to estimate the varying role of superspreading during the SARS-CoV-2 pandemic (Miyama et al. 2022; Guo et al. 2023). However, the literature has lacked a rigorous analytic exploration of how superspreading potential changes over time in a network epidemic by tracking the degrees of infected nodes over time.

In this work, we define three metrics of superspreading potential, measuring (1) the average number of contacts that infected nodes have, (2) the average number of those contacts which are susceptible, and (3) the average number of those susceptible contacts to which the infected node transmits the contagion. These metrics capture how the numbers of contacts, exposures, and transmissions associated with infected nodes change over time. We derive insights into how these metrics evolve in a continuous-time SIR epidemic over a static contact network with given degree distribution *K*, building on Miller and Volz’s edge-based model (Miller 2011; Miller et al. 2012).

Lastly, we discuss how these metrics are involved in inference methods for key epidemiological parameters from various sources of epidemic data. Importantly, our results show that inferred epidemiological parameters may differ greatly depending on the type of data available and how they are temporally aggregated.

### Overview of the Superspreading Metrics and Analytic Results

We formally define three metrics of superspreading potential, each of which is the mean of a distribution related to the properties of infected nodes at a given time. First, we define the *infected degree distribution* *X*(*t*) as the degree distribution of infected nodes at time *t*, which has mean *m*(*t*), variance *v*(*t*), moments $m_n(t)$, and mass function $p_k(t)$. Assuming that the initially seeded infected nodes are randomly chosen, the infected degree distribution will start out the same as the overall degree distribution of the network, $X(0)=K$ and $m(0)=\mu$. As we will show, *m*(*t*) first increases as *X*(*t*) approaches the “neighbor degree distribution” $K_{\text {n}}$ (degree distribution of random neighbors) due to high degree nodes getting primarily infected first, after which *m*(*t*) declines as high degree nodes recover and the infection moves to low degree nodes.

**Table 1:** Notation for the random variables (RV), probability mass functions (PMF), means, variances and moments associated with network degree, infected degree, effective degree, and secondary case distributions

Distribution	RV	PMF	mean	variance	*n*-th moment
Degree	*K*	*P*(*k*)	$\mu$	$\nu$	$\mu _n$
Infected degree	*X*(*t*)	$p_k(t)$	*m*(*t*)	*v*(*t*)	$m_n(t)$
Effective degree	*E*(*t*)	$p_{E,k}(t)$	$m_E(t)$	$v_E(t)$	$m_{E,n}(t)$
Secondary case	*Z*(*t*)	$p_{Z,k}(t)$	$m_Z(t)$	$v_Z(t)$	$m_{Z,n}(t)$

Second, we define the *effective degree distribution* *E*(*t*) as the distribution of the number of susceptible neighbors each infected node has at time *t* (named following Ref. Lindquist et al. 2011), which has mean $m_E(t)$, variance $v_E(t)$, moments $m_{E,n}(t)$, and mass function $p_{E,k}(t)$. This distribution captures the idea that while an infected node may have a large number of neighbors, its ability to transmit to many neighbors depends on it having a large number of susceptible neighbors, as these are the ones that it can actually infect. Naturally, $m_E(t)$ is always less than *m*(*t*), but it still starts at approximately $m_E(0)\approx \mu$ with the population starting out mostly susceptible. We show that $m_E(t)$ also has an initial increase in some parameter regimes, but not in others, followed also by a steady decline as susceptibility decreases.

**Table 2:** Summary of the analytic results derived in this paper concerning three metrics of superspreading at time *t* we define: the mean *m*(*t*) of the infected degree distribution, the mean $m_E(t)$ of the effective degree distribution, and the mean $m_Z(t)$ of the secondary case distribution. Here, $\beta$ is the transmission rate; $\gamma$ is the recovery rate; *I* is the infection prevalence; *J* is the instantaneous incidence; $\theta$ is the probability an edge has not yet transmitted infection; $\mathcal {R}_0$ is the basic reproduction number; $t_I$ is the peak time of *I*; and $\psi$ and $\varphi$ are the probability- and moment-generating functions, respectively, of the network’s degree distribution *K*

Variable	Equation	Peak value	
		(with $$\theta (0)\approx 1$$)	
*m*(*t*)	$\dot{m}=-\frac{J}{I}\big (m-\frac{\varphi ''(\log \theta )}{\varphi '(\log \theta )}\big )$	$\mu +\frac{\nu }{\mu }$	
$m_E(t)$	$\dot{m}_E=-\big (\frac{J}{I}+\beta -\frac{\psi ''(\theta )\dot{\theta }}{\psi '(\theta )}\big )m_E+\frac{J}{I}\frac{\psi ''(\theta )\theta }{\psi '(1)}$	$\max \{\mu +\frac{\nu }{\mu }-2,\mu \}$	
$m_Z(t)$	$m_Z(t)=\beta \frac{\psi ''(\theta (t))}{\psi '(\theta (t))}\int _t^\infty e^{-(\beta +\gamma )(\tau -t)}\frac{\psi '(\theta (\tau ))}{\psi '(1)}d\tau$	$<\mathcal {R}_0$ (if $\psi '$ log-convex)	

Variable	Peak time	Value in the limit $t\rightarrow \infty$	
	(with $\theta (0)\approx 1$)	if $\psi ''(\theta (\infty ))>\mu$	if $\psi ''(\theta (\infty ))\le \mu$
*m*(*t*)	$t_m<\big (\frac{1}{2}-\frac{\gamma }{4(\beta +\gamma )(\mathcal {R}_0-1)+2\gamma }\big )t_I$	$\ \frac{\varphi ''(\log \theta (\infty ))}{\varphi '(\log \theta (\infty ))}$	$>\frac{\varphi ''(\log \theta (\infty ))}{\varphi '(\log \theta (\infty ))}$
$m_E(t)$	0 if $\frac{\nu }{\mu }>2$, else $>0$ and $<t_m$	$\ \theta (\infty )\big (\frac{\psi ''(\theta (\infty ))}{\mu }-1\big )<\frac{\gamma }{\beta }$	0
$m_Z(t)$	0 (if $\psi '$ log-convex)	$\qquad \qquad \qquad \qquad \qquad =\frac{\beta }{\beta +\gamma }\frac{\psi ''(\theta (\infty ))}{\mu }<1$	

Third and lastly, we define the *secondary case distribution* *Z*(*t*) as the distribution of the number of secondary cases each newly infected node at time *t* will cause during its infectious period (named following Ref. Lloyd-Smith et al. 2005), which has mean $m_Z(t)$, variance $v_Z(t)$, moments $m_{Z,n}(t)$, and mass function $p_{Z,k}(t)$. While this is perhaps the most direct way of characterizing superspreading potential over time, it is also the least analytically tractable. However, we are still able to show that $m_Z(t)$ decreases monotonically at all times under certain assumptions, with no initial increase as with *m*(*t*) or $m_E(t)$. This difference occurs because *Z*(*t*) is concerned only with *newly* infected nodes at time *t*, whose degrees follow the neighbor degree distribution $K_{\text {n}}$ for small *t*. Conversely, *X*(*t*) and *E*(*t*) are concerned with all nodes currently infected at *t*, which for small *t* will still include some of the initial infections whose degrees follow the overall degree distribution *K*.

With these distributions rigorously defined and equations derived for them, we then investigate their means (and higher moments when possible), peak times, and limiting behavior. The notations for these distributions are summarized in Table 1 and the main analytic results are summarized in Table 2. Notably, we introduce the concept of the *superspreading peak*, defined as the peak of *m*(*t*), at the superspreading peak time $t_m$ when *m*(*t*) is maximized and the potential for superspreading could be considered to be at its highest. Interestingly, we show that the superspreading peak time $t_m$ is less than half the peak time $t_I$ of prevalence *I*(*t*), while $m_E(t)$ peaks before *m*(*t*) (and $m_Z(t)$ peaks even earlier at $t=0$). Thus, the potential for superspreading declines much earlier than the epidemic’s peak, meaning that much of the impact of superspreading may have already occurred by the time an outbreak has even reached epidemic proportions and become a serious threat.

### Overview of the Edge-Based Network Epidemic Model

Our analyses are based on Miller and Volz’s edge-based model (Miller 2011; Miller et al. 2012) for its simplicity, which we now briefly summarize. Miller and Volz describe a SIR epidemic in continuous time on a configuration model network (Newman 2018) as

1 $$\begin{aligned} \dot{\theta }&=-\beta \theta +\beta \frac{\psi '(\theta )}{\psi '(1)}+\gamma (1-\theta ) \end{aligned}$$

2 $$\begin{aligned} S&=\psi (\theta ) \end{aligned}$$

3 $$\begin{aligned} I&=1-S-R \end{aligned}$$

4 $$\begin{aligned} \dot{R}&=\gamma I, \end{aligned}$$

with *S*, *I*, and *R* representing the fraction of nodes susceptible, infected, and recovered, respectively ($S(t)+I(t)+R(t)=1$), $\beta$ is the transmission rate, $\gamma$ the recovery rate, and

5 $$\begin{aligned} \psi (x)=\langle x^K\rangle =\sum \limits _{k=0}^\infty P(k)x^k \end{aligned}$$

is the probability-generating function for the network’s degree distribution *K* with probability mass function *P*(*k*). The variable $0<\theta (t)<1$ represents the probability that an arbitrary neighbor of a focal susceptible node has not yet passed infection to that node by time *t* (either because the neighbor was never infected or the neighbor was infected but never transmitted along their connecting edge), which starts out at $\theta (0)\approx 1$ as we assume initial prevalence is small. Miller shows that an outbreak will occur if and only if

6 $$\begin{aligned} \mathcal {R}_0= \frac{\beta }{\beta +\gamma }\frac{\psi ''(1)}{\psi '(1)}>1, \end{aligned}$$

and then $\theta (t)$ will always be monotonically decreasing toward a final value $\theta (\infty )$. Since $\dot{\theta }(\infty )=0$, the quantity $\theta (\infty )$ must satisfy

7 $$\begin{aligned} \theta (\infty )=\frac{\gamma }{\beta +\gamma }+\frac{\beta }{\beta +\gamma }\frac{\psi '(\theta (\infty ))}{\psi '(1)}. \end{aligned}$$

From now on, $\theta (\infty )$ will refer to the unique solution to Eq. (7) between 0 and 1 exclusive, which Miller shows must exist. The function $\psi$ also has many useful properties: in this model, susceptibility is simply $S=\psi (\theta )$, while in general, $\psi '(1)=\langle K\rangle =\mu$ and $\psi ''(1)=\langle K(K-1)\rangle =\mu _2-\mu =\nu +\mu ^2-\mu$, where $\mu _n$ is the *n*-th moment of *K*, $\mu =\mu _1$ is its mean, and $\nu$ its variance (these notations are summarized in Table 1). It will also be helpful to define the instantaneous incidence of new infections

8 $$\begin{aligned} J=-\dot{S}=-\psi '(\theta )\dot{\theta }, \end{aligned}$$

and the moment-generating function of *K*

9 $$\begin{aligned} \varphi (y)=\langle e^{yK}\rangle =\sum \limits _{k=0}^\infty P(k)e^{yk} \end{aligned}$$

satisfying $$\varphi (\log x)=\psi (x)$$ and $$\varphi ^{(n)}(0)=\mu _n$$.

### Assumptions

Our results apply to any $\mathcal {R}_0>1$, while we ignore the case of $\mathcal {R}_0<1$ as this precludes the possibility of an outbreak occurring. We assume that the initial fraction of infected nodes is very small, which corresponds to an initial value of $\theta (0)$ very close to 1, as many of our results depend on this small initial infection limit. These initial infected nodes are also assumed to be chosen at random uniformly so that the degree distribution of initial infected nodes is the same as the degree distribution of the overall network (so that $m(0)=\mu$, though in the supplement Fig. S6 we show the effect of choosing initial infected nodes of different degrees by using alternative choices of *m*(0)). We also assume that at least the first three moments of *K* exist (excluding some power-law degree distributions from this analysis) so that $\psi '$, $\psi ''$, $\psi '''$ and $\varphi '$, $\varphi ''$, $\varphi '''$ are all well defined. We also assume that the network is static, large, and created by the configuration model, so that the network has negligible self-loops, multi-edges, degree correlations, clustering, and modularity (Newman 2018).

### Example Distributions and Simulations

**Fig. 1:**
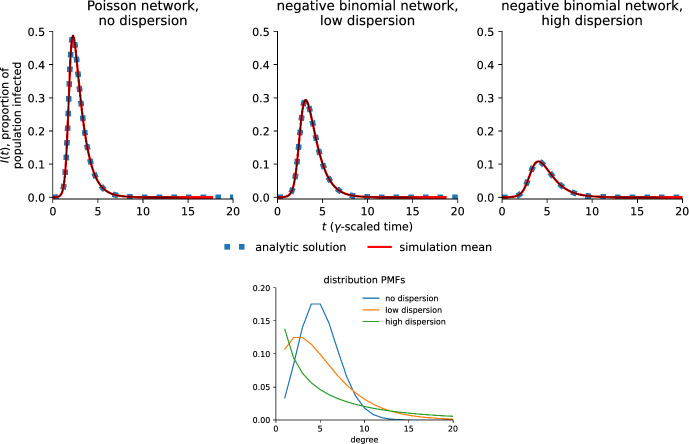
Trajectories of infection prevalence in the three networks we use as examples, each with 1 million nodes and mean degree 5: a Poisson network with no dispersion, a negative-binomial network with low dispersion (dispersion parameter $r=2.5$), and a negative-binomial network with high dispersion ($r=.5$). Red curves show the mean of 200 simulations (whose individual trajectories are also plotted by faint gray curves which coincide with the mean curve) while the blue dotted curves show the analytic solution provided by the edge-based model Eq. (1)–Eq. (4) (Miller 2011). We scale time *t* by the infection recovery rate $\gamma$, meaning time is in units of average infectious period. The bottom plot shows the probability mass functions (PMFs) for each of the distributions we examine in this and the rest of the plots in this paper. We also show infection prevalence but in log scale in the supplement Fig. S5, to showcase that the epidemic reaches a highly stochastic phase once the number of infected nodes reaches about 10 (color figure online)

As examples, we investigate epidemics on networks with three different degree distributions: (1) *K* is Poisson distributed, with no dispersion; (2) *K* is negative-binomially distributed with low dispersion (dispersion parameter $r=2.5$); and (3) *K* is negative-binomially distributed with high dispersion ($r=.5$). In Fig. 1, we show what the probability mass functions and epidemic trajectories look like for each of these three network degree distributions. Higher dispersion creates smaller outbreaks as the superspreaders are more scarce and more likely to become infected early in the epidemic, leaving only low degree nodes to be infected later (Miller et al. 2012).

We employ negative binomial distributions (and Poisson distributions in the limiting case of no dispersion) as they have been found to be good fits to empirical superspreading event (Lloyd-Smith et al. 2005) and allow exact edge-based description (Kiss et al. 2023). For all three, we fix the mean degree $\mu =5$ and the disease’s basic reproduction number $\mathcal {R}_0=3$. From there, $\beta$ can be calculated from Eq. (6), while $\gamma$ can be thought of as a time-scale constant and set arbitrarily to 1 (we do so and thus refer to *t* as “$\gamma$-scaled time”).

We construct networks for each of these three degree distributions and with 1 million nodes each using the configuration model and removing self-loops and multi-edges. On each of these three networks, we use code originally written for the “Epidemics on Networks” Python package (Miller and Ting 2019) to run 200 simulations, each starting with a random set of 100 infections out of the 1 million nodes.

## The Infected Degree Distribution *X*(*t*)

To derive the infected degree distribution, it will be helpful to have equations for how overall prevalence *I*(*t*) changes over time as well as $I_k(t)$, which we define to be the prevalence of infection among nodes of degree *k*. The first of these follows immediately from differentiating Eq. (3) to get

10 $$\begin{aligned} \dot{I}=-\psi '(\theta )\dot{\theta }-\gamma I. \end{aligned}$$

For degree *k* nodes, and for each of their *k* neighbors, $\theta$ is the probability that the neighbor has not yet passed infection to the node, so that a degree *k* node will be susceptible with probability $\theta ^k$. From this logic, we get an analog to the original model but for degree *k* nodes only:

11 $$\begin{aligned} S_k&=\theta ^k \end{aligned}$$

12 $$\begin{aligned} I_k&=1-S_k-R_k \end{aligned}$$

13 $$\begin{aligned} \dot{R}_k&=\gamma I_k \end{aligned}$$

and

14 $$\begin{aligned} \dot{I}_k=-k\theta ^{k-1}\dot{\theta }-\gamma I_k, \end{aligned}$$

with the dynamics of $\theta$ still described by Eq. (1).

By Bayes’ law, the probability that an infected node is of degree *k* equals

15 $$\begin{aligned} p_k=\frac{P(k)I_k}{I}, \end{aligned}$$

which we can differentiate to get $\dot{p}_k=P(k)\frac{I\dot{I}_k-I_k\dot{I}}{I^2}$, simplifying to

16 $$\begin{aligned} \dot{p}_k=-\frac{J}{I}\left( p_k-\frac{P(k)k\theta ^{k-1}}{\psi '(\theta )}\right) , \end{aligned}$$

where again $J=-\psi '(\theta )\dot{\theta }$ is the instantaneous incidence of new infections. The $p_k$ represent the mass function for the infected degree distribution *X*(*t*), which starts out equal to the network degree distribution so that $p_k(0)=P(k)$.

Alternately, we can express $\dot{p}_k$ in terms of the degree distribution’s moment-generating function $\varphi$, which instead yields

17 $$\begin{aligned} \dot{p}_k=-\frac{J}{I}\left( p_k-\frac{P(k)k\theta ^k}{\varphi '(\log \theta )}\right) . \end{aligned}$$

From this formulation, succinct differential equations for the infected degree distribution’s moments easily follow.

### Theorem 1

Assume $\mu _{n+1}$, the $(n+1)$-th moment of the network degree distribution, exists. Then the *n*-th moment of the infected degree distribution *X*(*t*) exists and satisfies

18 $$\begin{aligned} \dot{m}_n=-\frac{J}{I}\left( m_n-\frac{\varphi ^{(n+1)}(\log \theta )}{\varphi '(\log \theta )}\right) , \end{aligned}$$

with initial value of $m_n$ given by

19 $$\begin{aligned} m_n(0)=\mu _n. \end{aligned}$$

### Proof

Note that, since $\mu _{n+1}$ exists, $\varphi ^{(j)}(\log \theta )$ will also exist for any $j\le n{+}1$ and $0{<}\theta {<}1$, as $\varphi ^{(j)}(\log \theta ){=}\langle K^j\theta ^K\rangle \le \langle K^{n+1}\rangle {=}\mu _{n+1}$. Eq. (18) then follows from Eq. (17) as

$$\begin{aligned} \dot{m}_n&= \sum _{k=0}^\infty \dot{p}_k k^n\\&= -\frac{J}{I}\left( \sum _{k=0}^\infty p_kk^n-\sum _{k=0}^\infty \frac{P(k)k^{n+1}\theta ^k}{\varphi '(\log \theta )}\right) \\&= -\frac{J}{I}\left( m_n-\frac{\varphi ^{(n+1)}(\log \theta )}{\varphi '(\log \theta )}\right) . \end{aligned}$$

And since the initial infected degree distribution and the network degree distribution are equal, so are their moments, such that $m_n(0)=\mu _n$. $\square$

**Fig. 2:**
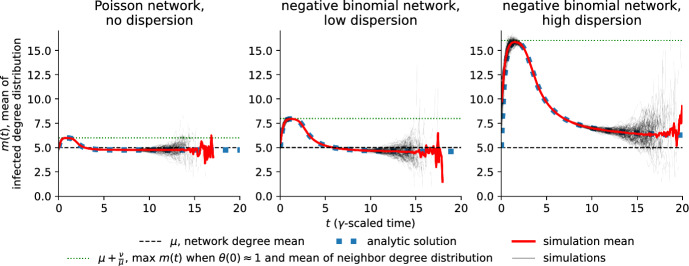
Trajectories of the infected degree distribution’s mean *m*(*t*) for three different network degree distributions. Red curves show the mean of 200 simulations (whose individual trajectories are also plotted by faint gray curves) while the blue dotted curves show the analytic solution from Eq. (20). Black dashed lines show the mean $\mu$ of the network degree distribution and green dotted lines show the mean $\mu +\frac{\nu }{\mu }$ of the neighbor degree distribution (color figure online)

Intuitively, Eq. (18) says that $m_n(t)$ is continuously being attracted to the moving target $\frac{\varphi ^{(n+1)}(\log \theta (t))}{\varphi '(\log \theta (t))}$ at a rate proportional to $\frac{J(t)}{I(t)}$, which we call the *infection turnover rate*. This is the relative rate at which current infections are replaced with new ones, thus also controlling the rate at which the degree distribution of infected nodes changes. Before continuing, it will be helpful to establish the behavior of this infection turnover rate, with a lemma we prove in the supplement.

### Lemma 2

The following results are true for the infection turnover rate $\frac{J(t)}{I(t)}$ when $\theta (0)$ is sufficiently close to 1:

1. $$\frac{J(t)}{I(t)}>\gamma $$ while $$\dot{J}(t)\ge 0$$.
2. $\frac{J(t)}{I(t)}<\beta \left( \frac{\psi ''(1)}{\psi '(1)}-1\right)$ always, and $\frac{J(t)}{I(t)}$ can be made to stay arbitrarily close to $\beta \left( \frac{\psi ''(1)}{\psi '(1)}-1\right)$ for an arbitrary amount of time while $\theta (t)\approx 1$ by choosing $\theta (0)$ sufficiently close to 1.
3. $$\lim \limits _{t\rightarrow \infty }\frac{J(t)}{I(t)}=\max \left\{ \beta \left( \frac{\psi ''(\theta (\infty ))}{\psi '(1)}-1\right) ,0\right\} $$.

We illustrate this behavior of infection turnover rate $\frac{J(t)}{I(t)}$ in supplementary Fig. S4.

Now, we are specifically interested in the mean and variance of the infected degree distribution, which by Theorem 1 for $n=1$ satisfies

20 $$\begin{aligned} \dot{m}=\frac{J}{I}\left( m-\frac{\varphi ''(\log \theta )}{\varphi '(\log \theta )}\right) \end{aligned}$$

with $m(0)=\mu$, and $v(t)=\mu _2(t)-\mu (t)^2$ with $\mu _2(t)$ also defined by Theorem 1. We show the trajectories of *m*(*t*) in Fig. 2 and the trajectories of *v*(*t*) in supplementary Fig. S1.

The mean infected degree *m*(*t*) is seen to first increase from the mean of the network degree distribution $\mu$, peak at approximately the mean of the neighbor degree distribution $\mu +\frac{\nu }{\mu }$, then decline monotonically. This is in fact true of all moments of *X*(*t*): $m_n(t)$ starts out at the *n*-th moment of the network degree distribution $\mu _n$, peaks at approximately the *n*-th moment of the neighbor degree distribution $\frac{\varphi ^{(n+1)}(0)}{\varphi (0)}$, then declines monotonically. We call this peak of *m*(*t*), when the infected degree distribution is approximately equal to the neighbor degree distribution, the *superspreading peak*. This notion can be formalized by the following result.

### Theorem 3

In the limit as $\theta (0)\approx 1$, the infected degree distribution *X*(*t*) will approach the neighbor degree distribution $K_{\text {n}}$ at some time *t* (which may change with $\theta (0)$ as it approaches 1). Specifically, for any small $\varepsilon >0$ and $k\in \mathbb {N}$, there exist a $\theta (0)$ and time $t_\varepsilon$ for which $\left\lvert p_j(t_\varepsilon )-\frac{P(k)k}{\mu } \right\rvert <\varepsilon$ for all $j\le k$.

### Proof

We provide an outline of the proof here and complete the details in the supplement. By Eq. (17), $p_k(t)$ is constantly moving toward the moving target $\frac{P(k)k\theta (t)^k}{\varphi '(\log \theta (t))}$ at rate proportional to the infection turnover rate $\frac{J(t)}{I(t)}$. Conversely, $\theta (t)$ decreases monotonically and is bounded below by

21 $$\begin{aligned} \theta (t)>1-(1-\theta (0))e^{(\beta +\gamma )(\mathcal {R}_0-1)t}\ \text { for all }t>0, \end{aligned}$$

which follows from Eq. (1) via

22 $$\begin{aligned} \frac{d}{dt}\log (1-\theta (t))=(\beta +\gamma )(\mathcal {R}_0-1)-\frac{\beta }{\psi '(1)}\left( \psi ''(1)-\frac{\psi '(1)-\psi '(\theta (t))}{1-\theta (t)}\right) \end{aligned}$$

and from the convexity of $\psi '$ (since $\psi '''$ is assumed to exist and is positive). Thus, for any fixed value $\theta _J>\theta (\infty )$, by setting $\theta (0)$ sufficiently close to 1 we can ensure that there is a time $t_J$ for which $\theta (t_J)=\theta _J$ and this time can be made arbitrarily large by setting $\theta (0)$ sufficiently close to 1. Specifically, we choose $t_J>0$ to be the time at which incidence *J*(*t*) peaks, which we show must occur and that $\theta _J=\theta (t_J)$ is constant regardless of initial condition. Furthermore, Lemma 2 tells us that $\frac{J(t)}{I(t)}>\gamma$ for all $t\le t_J$. With this constant positive lower bound on the infection turnover rate $\frac{J(t)}{I(t)}$ for $t\le t_J$ and with the ability to make $t_J$ arbitrarily large by choosing $\theta (0)$ sufficiently close to 1, we can then choose a $\theta (0)$ that will keep $\theta (1)$ close to 1 and $\frac{J(t)}{I(t)}>\gamma$ long enough for $p_j(t_J)$ to reach sufficiently close to $\frac{P(k)k}{\mu }$ for all $j\le k$. $\square$

The peak values of *m*(*t*) and higher moments $m_n(t)$ come as a direct corollary.

### Corollary 4

In the limit as $\theta (0)\rightarrow 1$, $m_n(t)$ peaks at value

23 $$\begin{aligned} \lim \limits _{\theta (0)\rightarrow 1}\max \limits _{t\ge 0}m_n(t)=\frac{\varphi ^{(n+1)}(0)}{\varphi '(0)}. \end{aligned}$$

In particular,

24 $$\begin{aligned} \lim \limits _{\theta (0)\rightarrow 1}\max \limits _{t\ge 0}m(t)=\mu +\frac{\nu }{\mu }. \end{aligned}$$

We are also interested in the time $t_m$ it takes for the mean infected degree *m*(*t*) to peak, called the *superspreading peak time*, and its relation to the peak time $t_I$ of prevalence *I*(*t*). Interestingly, we find that the peak time for *m*(*t*) is always less than half the peak time of *I*(*t*) when $\theta (0)$ is sufficiently close to 1, suggesting that the potential for superspreading is already diminished by the time infections in an epidemic have taken off. In the next result, we provide an upper bound on the ratio $\frac{t_m}{t_I}$ in the limit of $\theta (0)\rightarrow 1$ that is even tighter than $\frac{1}{2}$, while noting that in this limit $\frac{t_m}{t_I}$ stays greater than 0 despite $t_I$ going to $\infty$.

### Theorem 5

If $\theta (0)$ is sufficiently close to 1, then *m*(*t*) and *I*(*t*) will both peak at times $t_m>0$ and $t_I>0$ respectively. And as $\theta (0)$ approaches 1 both $t_m$ and $t_I$ will diverge to $\infty$ while

25 $$\begin{aligned} 0<\lim _{\theta (0)\rightarrow 1}\frac{t_m}{t_I}<\frac{1}{2}-\frac{\gamma }{4(\beta +\gamma )(\mathcal {R}_0-1)+2\gamma }. \end{aligned}$$

### Proof

We provide an outline of the proof here and complete the details in the supplement. We have already shown in Theorem 3 that $t_m$ exists and that $t_J$ goes to infinity as $\theta (0)$ approaches 1, and we show in the supplement that $t_m$ also goes to infinity. And the peak $t_I>0$ of *I*(*t*) also exists from the fact that *I*(0) is close to zero and $\mathcal {R}_0>1$ by assumption (Miller 2011). Furthermore, $\lim \limits _{\theta (0)\rightarrow 1}\frac{t_I}{t_J}=1$, as we show in the supplement. Thus, we can replace $t_I$ with $t_J$ in Eq. (25), which greatly simplifies the analysis since, as we have previously mentioned, $\theta (t_J)=\theta _J$ is always constant regardless of initial conditions.

We now consider $1-\theta (t)$ and bound it above and below by exponential functions for $t\le t_J$. Above, it is bounded by an exponential with rate $\lambda _1=(\beta +\gamma )(\mathcal {R}_0-1)$ from Eq. (21). And below it is bounded by an exponential with rate $\lambda _2=\lambda _1-\frac{\beta }{\psi '(1)}\left( \psi ''(1)-\frac{\psi '(1)-\psi '(\theta _J)}{1-\theta _J}\right)$ from Eq. (22) and since $\frac{\psi '(1)-\psi '(x)}{1-x}$ is increasing for $x<1$ from the convexity of $\psi '$. Since $\lambda _1>\lambda _2>0$, as we prove, then

26 $$\begin{aligned} 0<\frac{1}{\lambda _1}<\frac{t_J}{\log \frac{1}{1-\theta (0)}+\log (1-\theta _J)}<\frac{1}{\lambda _2}. \end{aligned}$$

To derive $t_m$, note that the $\psi ''(1)-\frac{\psi '(1)-\psi '(\theta (t))}{1-\theta (t)}$ term in Eq. (22) vanishes uniformly across all $t\le t_m$ as $\theta (0)\rightarrow 1$ (since we have shown that $\lim \limits _{\theta (0)\rightarrow 1}\theta (t_m)=1$), especially as compared to $\log (1-\theta (t))$ whose magnitude becomes arbitrary large for $t\le t_m$ as $\theta (0)\rightarrow 1$. Thus, the inequality in Eq. (21) approaches equality uniformly across all $t\le t_m$ in the limit as $\theta (0)\rightarrow 1$, and

27 $$\begin{aligned} \lim \limits _{\theta (0)\rightarrow 1}\dot{m}(t)=-\frac{J(t)}{I(t)}\left( m-\big (f(1)-f'(1)(1-\theta (0))e^{(\beta +\gamma )(\mathcal {R}_0-1)t}\big )\right) \text { for all }t\le t_m \end{aligned}$$

converges uniformly across $t\le t_m$, with $f(x)=\frac{\varphi ''(\log x)}{\varphi '(\log x)}$. From this limiting behavior for *m*(*t*) and by Lemma 2, we can show

28 $$\begin{aligned} \lim \limits _{\theta (0)\rightarrow 1}\frac{t_m}{\log \frac{1}{1-\theta (0)}}=\frac{1}{2\lambda _1+\gamma }. \end{aligned}$$

Finally, as $\theta (0)\rightarrow 1$ and $\log \frac{1}{1-\theta (0)}\rightarrow \infty$, we see that $t_m$ diverges, and dividing Eq. (28) by Eq. (26) then replacing $t_J$ with $t_I$ in the limit as $\theta (0)\rightarrow 1$ gives the desired result. $\square$

**Fig. 3:**
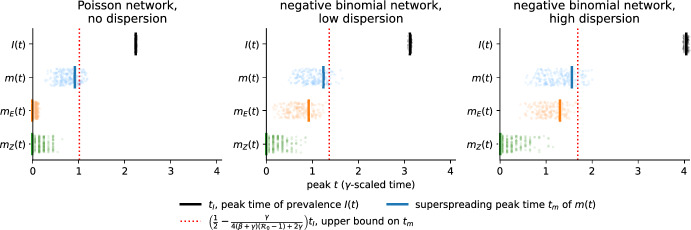
Peak times of *I*(*t*), *m*(*t*), $m_E(t)$, and $m_Z(t)$ for three different network degree distributions, and from both simulation (faint points in background, binned into multiples of .1 for $m_Z(t)$) and analytic (solid lines) trajectories. The dashed red line shows the upper bound on the superspreading peak time $t_m$ of *m*(*t*) given by Theorem 5, demonstrating that all three superspreading metrics we consider peak sooner than half the time it takes for infection prevalence to peak. When measuring simulation peak times, we ignore the highly stochastic period at the end of the epidemics where there are very few infections and these metrics may have wild fluctuations (as visible in Figs. 2, 5, 6, and S5) (color figure online)

In Fig. 3, we show how the superspreading peak time $t_m$ compares to this upper bound and to the prevalence peak time $t_I$.

Lastly, we aim to find the limiting behavior of the infected degree distribution as $t\rightarrow \infty$. Eq. (18) shows that *m*(*t*) will eventually move toward the value $\frac{\varphi ^{(n+1)}(\theta (\infty ))}{\varphi '(\theta (\infty ))}$, but this will only be reached in the limit of $t\rightarrow \infty$ when the infection turnover rate $\frac{J(t)}{I(t)}$ stays greater than 0, which we know occurs if and only if $\psi ''(\theta (\infty ))>\mu$ by Lemma 2. Similarly, Eq. (17) shows that $p_k(t)$ will reach $\frac{P(k)k\theta (\infty )^k}{\varphi '(\log \theta (\infty ))}$ as $t\rightarrow \infty$ if and only if $\psi ''(\theta (\infty ))>\mu$. This immediately gives the result

### Theorem 6

If $$\psi ''(\theta (\infty ))>\mu $$, then

$$\begin{aligned} \lim \limits _{t\rightarrow \infty }m_n(t)=\frac{\varphi ^{(n+1)}(\theta (\infty ))}{\varphi '(\theta (\infty ))} \end{aligned}$$

for any *n* for which $\mu _{n+1}$, the $(n+1)$-th moment of the network degree distribution, exists; and

$$\begin{aligned} \lim \limits _{t\rightarrow \infty }p_k(t)=\frac{P(k)k\theta (\infty )^k}{\varphi '(\log \theta (\infty ))} \end{aligned}$$

for all *k*.

Otherwise, if $\psi ''(\theta (\infty ))\le \mu$, then

$$\begin{aligned} \lim \limits _{t\rightarrow \infty }m_n(t)>\frac{\varphi ^{(n+1)}(\theta (\infty ))}{\varphi '(\theta (\infty ))} \end{aligned}$$

for any *n* for which $\mu _{n+1}$ exists.

Before moving on to deriving the effective degree distribution and the secondary case distribution, we provide the examples of infected degree distributions at the superspreading peak and at the end of the epidemic for Poisson and negative binomial networks. These follow directly from Theorems 3 and 6.

**Fig. 4:**
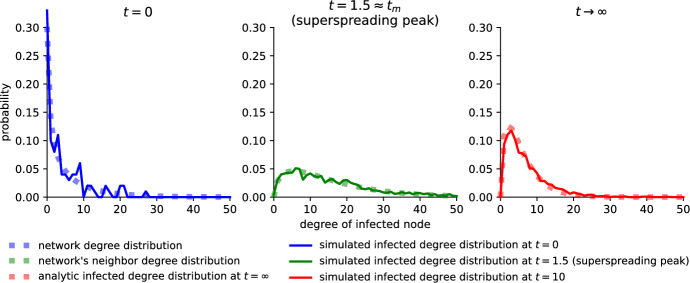
The infected degree distribution at the beginning, superspreading peak, and end of an epidemic on a negative binomial distribution with high dispersion (dispersion parameter $r=.5$). Solid lines showing infected degree distributions from a single simulation and dashed lines showing the analytic infected degree distributions given by the network degree distribution *K* at $t=0$, by Eq. (31) at the superspreading peak, and by Eq. (32) for $t\rightarrow \infty$ (Color Figure Online)

### Examples

If the network degree distribution is Poisson with rate parameter $\lambda$ ($K\sim {{\,\textrm{Pois}\,}}(\lambda )$), then at the superspreading peak $t_m$, the infected degree distribution *X* follows

29 $$\begin{aligned} X(t_m)-1\approx {{\,\textrm{Pois}\,}}(\lambda ). \end{aligned}$$

If $\psi ''(\theta (\infty ))>\mu$, then as $t\rightarrow \infty$,

30 $$\begin{aligned} X(\infty )-1\sim {{\,\textrm{Pois}\,}}\big (\theta (\infty )\lambda \big ). \end{aligned}$$

If the network degree distribution is negative binomial with dispersion parameter *r* and success probability parameter *p* ($K\sim {{\,\textrm{NB}\,}}(r,p)$), then at the superspreading peak $t_m$,

31 $$\begin{aligned} X(t_m)-1\approx {{\,\textrm{NB}\,}}(r+1,p). \end{aligned}$$

If $\psi ''(\theta (\infty ))>\mu$, then as $t\rightarrow \infty$,

32 $$\begin{aligned} X(\infty )-1\sim {{\,\textrm{NB}\,}}\big (r+1,1-\theta (\infty )(1-p)\big ). \end{aligned}$$

We demonstrate this result in Fig. 4 for the negative binomial distribution with high dispersion (dispersion parameter $r=.5$, as this is the only network we examine that satisfies $\psi ''(\theta (\infty ))>\mu$, as shown by supplementary Fig. S4).

## The Effective Degree Distribution *E*(*t*)

Since some of an infected node’s neighbors may be susceptible at any time, and thus unable to have infection transmitted to them, it is important to consider not only how many neighbors an infected node has, but also how many of those neighbors are susceptible. We call the distribution of the number susceptible neighbors that infected nodes have at time *t* the *effective degree distribution* *E*(*t*). By definition, $E(t)\le X(t)$.

Deriving this distribution is less straightforward, but can be done with the help of new variables $J_j(t)$, the instantaneous incidence of newly infected degree-*j* nodes at time *t*, and $H_j(t)$, the probability that a degree *j* node is currently infected at time *t* and has not transmitted infection to a specific, arbitrary neighbor. Just like with *J*(*t*), $J_j(t)$ can easily be derived as

33 $$\begin{aligned} J_j=-\dot{S}_j=-j\theta ^{j-1}\dot{\theta }. \end{aligned}$$

And just as $\dot{I}_j=J_j-\gamma I_j$ for *I*(*t*) since degree *j* nodes become infected at rate $J_j$ and stop being infected at rate $\gamma$, we now have

34 $$\begin{aligned} \dot{H}_j=J_j-(\beta +\gamma )H_j,\ H_j(0)=I(0), \end{aligned}$$

since a node stops having the property “infected but has not yet transmitted infection to an arbitrary neighbor” at rate $\beta +\gamma$ (by either infecting the neighbor or recovering). Initially, $H_j(0)=I_j(0)=I(0)$ since there has been no time yet for any transmissions to happen yet at $t=0$.

To figure out how many susceptible neighbors an infected node of degree *j* has at time *t*, we must consider two cases: (1) the node was an initial infected node or (2) it was not an initial infected node. The probability that a degree-*j* infected node is of the first case is $\frac{P(j)e^{-\gamma t}I(0)}{I(t)}$ by Bayes’ law, since $e^{-\gamma t}I(0)$ is the probability that an initial infected node is still infected by time *t*. The probability that a degree-*j* node is of the second case is thus $p_j-\frac{P(j)e^{-\gamma t}I(0)}{I(t)}=\frac{P(j)(I_j(t)-e^{-\gamma t}I(0))}{I(t)}$.

For the first case, consider an initial infected node of degree *j* which is still infected at time *t*. The probability that one of its neighbors with degree *l* has not yet had infection transmitted to it by any of its other $l-1$ neighbors is $\theta (t)^{l-1}$. Thus, the probability that a degree-*l* neighbor is still susceptible is equal to $\theta (t)^{l-1}$ times the probability that the initial infected node has not transmitted infection to this neighbor, which is $e^{-\beta t}$. Then considering that a neighbor will be degree *l* with probability $\frac{P(l)l}{\psi '(1)}$ according to the neighbor degree distribution, the probability that an arbitrary neighbor of the initial infected node is susceptible at time *t* is $\sum _{l=0}^\infty \frac{P(l)l\theta (t)^{l-1}}{\psi '(1)}e^{-\beta t}=\frac{\psi '(\theta (t))}{\psi '(1)}e^{-\beta t}$, and the probability that the initial infected node has exactly *k* susceptible neighbors is

35 $$\begin{aligned} \eta _{1,j,k}(t)=\left( {\begin{array}{c}j\\ k\end{array}}\right) \left( \frac{\psi '(\theta (t))}{\psi '(1)}e^{-\beta t}\right) ^k\left( 1-\frac{\psi '(\theta (t))}{\psi '(1)}e^{-\beta t}\right) ^{j-k}. \end{aligned}$$

For the second case, consider an infected node of degree *j* at time *t* which was not infected initially. The calculation is similar to the first case, except now the infected node can only have a maximum of $j-1$ susceptible neighbors, instead of *j* as before, since one of the infected node’s neighbors must have infected it. And now the probability that this non-initial infected node has not yet transmitted infection to an arbitrary neighbor, given its non-initial infected state, is $\frac{H_j(t)-e^{-(\beta +\gamma )t}I(0)}{I_j(t)-e^{-\gamma t}I(0)}$, with $H_j(t)-e^{-(\beta +\gamma )tI(0)}$ representing the probability that a degree *j* node is infected but has not yet transmitted to an arbitrary neighbor and was not an initial infected. From this, we get the probability that this non-initial infected node has exactly *k* susceptible neighbors to be

36 $$\begin{aligned} \begin{aligned} \eta _{2,j,k}(t)=&\left( {\begin{array}{c}j-1\\ k\end{array}}\right) \left( \frac{\psi '(\theta (t))}{\psi '(1)}\frac{H_j(t)-e^{-(\beta +\gamma )t}I(0)}{I_j(t)-e^{-\gamma t}I(0)}\right) ^k\times \\&\quad \left( 1-\frac{\psi '(\theta (t))}{\psi '(1)}\frac{H_j(t)-e^{-(\beta +\gamma )t}I(0)}{I_j(t)-e^{-\gamma t}I(0)}\right) ^{j-1-k}. \end{aligned} \end{aligned}$$

**Fig. 5:**
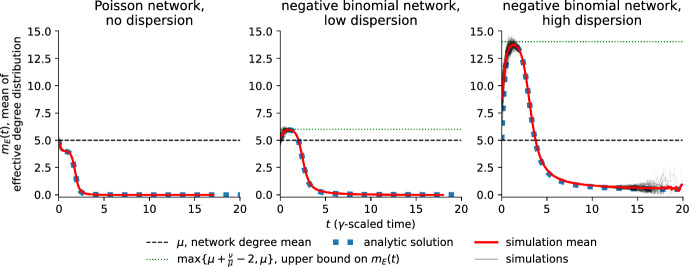
Trajectories of the effective degree distribution’s mean $m_E(t)$ for three different network degree distributions. Red curves show the mean of 200 simulations (whose individual trajectories are also plotted by faint gray curves) while the blue dotted curves show the analytic solution from Eq. (39). Black dashed lines show the mean $\mu$ of the network degree distribution and green dotted lines show the upper bound $\max \{\mu +\frac{\nu }{\mu }-2,\mu \}$ provided by Theorem 8 (color figure online)

Putting this all together, we arrive at the mass function $p_{E,k}(t)$ for the effective degree distribution *E*(*t*):

37 $$\begin{aligned} p_{E,k}(t)=\sum _{j=k}^\infty \frac{P(j)e^{-\gamma t}I(0)}{I(t)}\eta _{1,j,k}(t)+\sum _{j=k+1}^\infty \frac{P(j)(I_j(t)-e^{-\gamma t}I(0))}{I(t)}\eta _{2,j,k}(t). \end{aligned}$$

While this expression is not particularly nice, the mean $m_E(t)=\sum _{k=0}^\infty p_{E,k}(t)$ of *E*(*t*) simplifies to

38 $$\begin{aligned} m_E(t)=\frac{\psi '(\theta (t))}{\psi '(1)}\left[ \sum _{j=1}^\infty P(j)(j-1)\frac{H_j(t)}{I(t)} + \frac{e^{-(\beta +\gamma )t}I(0)}{I(t)}(1-P(0))\right] \end{aligned}$$

where *P*(0) is the probability a node in the network has degree 0, as the sums of the binomial terms simplify to the expectations of binomial random variables. However, we can do even better by considering how $m_E(t)$ changes over time, expressing it with the following differential equation:

### Theorem 7

The mean $m_E(t)$ of the effective degree distribution *E*(*t*) satisfies

39 $$\begin{aligned} \dot{m}_E=-\left( \frac{J}{I}+\beta -\frac{\psi ''(\theta )\dot{\theta }}{\psi '(\theta )}\right) m_E+\frac{-\psi '(\theta )\dot{\theta }}{I}\frac{\psi ''(\theta )\theta }{\psi '(1)}, \end{aligned}$$

with initial value given by

40 $$\begin{aligned} m_E(0)=\psi '(\theta (0))\approx \mu . \end{aligned}$$

### Proof

Eq. (39) follows from differentiating Eq. (38). And Eq. (40) follows since $H_j(0)=I(0)$ for all *j*, so $m_E(0)=\frac{\psi '(\theta (t))}{\psi '(1)}\left[ \sum _{j=1}^\infty P(j)(j-1) +(1-P(0))\right] =\psi '(\theta (0))$, which is approximately $\psi '(1)=\mu$ for $\theta (0)\approx 1$. $\square$

The higher moments of *E*(*t*) do not yield such nice differential equations, but we do provide a formula for the second moment $m_{E,2}(t)$ in the supplement, from which the variance can be calculated as $v_E(t)=m_{E,2}(t)-m_E(t)^2$. We show the trajectories of $m_E(t)$ in Fig. 5 and the trajectories of $v_E(t)$ in supplementary Fig. S2.

The next result focuses on the peak behavior of $m_E(t)$, which we show occurs at $t=0$ in networks where $\frac{\nu }{\mu }\le 2$ but occurs later when $\frac{\nu }{\mu }>\mu$. In general, $\max \{\mu +\frac{\nu }{\mu }-2,\mu \}$ is an upper bound to $m_E(t)$, which $m_E(t)$ achieves in the limit as $\theta (0)\rightarrow 1$, and the peak time of $m_E(t)$ always occurs before the superspreading peak time $t_m$ of *m*(*t*).

### Theorem 8

If $\frac{\nu }{\mu }\le 2$, then in the limit as $\theta (0)\rightarrow 1$, $m_E(t)$ will peak at time $t=0$ and at value $m_E(0)=\mu$. If $\frac{\nu }{\mu }>2$, then in the limit as $\theta (0)\rightarrow 1$, $m_E(t)$ will peak at a time greater than 0 but less than the superspreading peak time $t_m$ of *m*(*t*) and at the value $\mu +\frac{\nu }{\mu }-2$. Thus,

41 $$\begin{aligned} \lim \limits _{\theta (0)\rightarrow 1}\max \limits _{t\ge 0} m_E(t)=\max \left\{ \mu +\frac{\nu }{\mu }-2,\mu \right\} . \end{aligned}$$

The peak time of $m_E(t)$ is less than $t_m$ if $\theta (0)$ is sufficiently close to 1, and will occur at $t=0$ if $\frac{\nu }{\mu }\le 2$. And the value of $m_E(t)$ at its peak is bounded above by

42 $$\begin{aligned} \max \limits _{t\ge 0}m_E(t)<\max \{\mu +\frac{\nu }{\mu }-2,\mu \}. \end{aligned}$$

### Proof

Reformatting Eq. (39) as

43 $$\begin{aligned} \dot{m}_E=-\left( \frac{J}{I}+\beta -\frac{\psi ''(\theta )}{\psi '(\theta )}\dot{\theta }\right) \left( m_E-\frac{J/I}{J/I+\beta -\frac{\psi ''(\theta )}{\psi '(\theta )}\dot{\theta }}\frac{\psi ''(\theta )\theta }{\psi '(1)}\right) \end{aligned}$$

shows that $m_E$ is constantly attracted toward the moving target $\frac{J/I}{J/I+\beta -\frac{\psi ''(\theta )}{\psi '(\theta )}\dot{\theta }}\frac{\psi ''(\theta )\theta }{\psi '(1)}$ at rate $\frac{J}{I}+\beta -\frac{\psi ''(\theta )}{\psi '(\theta )}\dot{\theta }$. Since $\dot{\theta }<0$ and $\frac{J}{I}<\beta \left( \frac{\psi ''(1)}{\psi '(1)}-1\right)$ by Lemma 2, this moving target will always be bounded above by

44 $$\begin{aligned} \frac{J/I}{J/I+\beta -\frac{\psi ''(\theta )}{\psi '(\theta )}\dot{\theta }}\frac{\psi ''(\theta )\theta }{\psi '(1)}<\left( 1-\frac{\beta }{J/I+\beta }\right) \frac{\psi ''(1)}{\psi '(1)}<\frac{\psi ''(1)}{\psi '(1)}-1=\mu +\frac{\nu }{\mu }-2. \end{aligned}$$

If $\frac{\nu }{\mu }\le 2$, then the moving target $\frac{J/I}{J/I+\beta -\frac{\psi ''(\theta )}{\psi '(\theta )}\dot{\theta }}\frac{\psi ''(\theta )\theta }{\psi '(1)}$ will always be bounded above by $\mu =\lim \limits _{\theta (0)\rightarrow 1}m_E(0)$, and so in the limit as $\theta (0)\rightarrow 1$, $m_E(t)$ will peak at $t=0$ at value $m_E(0)=\mu$.

Now assume that $\frac{\nu }{\mu }>2$. Lemma 2 gives us that $\frac{J}{I}$ approaches $\beta \left( \frac{\psi ''(1)}{\psi '(1)}-1\right)$ from below at the beginning and can be made to stay arbitrarily close to this value for arbitrarily long while $\theta (t)\approx 1$ by choosing $\theta (0)$ sufficiently close 1. Thus, in the limit as $\theta (0)\rightarrow 1$, when calculating the target $m_E(t)$ moves toward, we can consider $\theta \approx 1$ and $\dot{\theta }\approx 0$ as constants, and $\frac{J}{I}$ as being less than but approaching $\beta \left( \frac{\psi ''(1)}{\psi '(1)}-1\right)$, so that Eq. (44) will always be true but in the limit as $\theta (0)\rightarrow 1$ the inequalities in Eq. (44) will tend toward equalities. $\square$

This result is reflected in Fig. 5, which shows that $m_E(t)$ always stays under this upper bound of $\max \{\mu +\frac{\nu }{\mu }-2,\mu \}$, and in Fig. 3, which shows that the peak times of $m_E(t)$ are always less than the superspreading peak times $t_m$ (and consequently also bounded above by half the prevalence peak time $t_I$). Furthermore, in the case of the Poisson network where $\frac{\nu }{\mu }=1\le 2$, we see that $m_E(t)$ does indeed peak at $t=0$, while in the other two cases where $\frac{\nu }{\mu }>2$, we see that $m_E(t)$ peaks later and at a value approximately equal to $\mu +\frac{\nu }{\mu }-2$. This peak value of $\mu +\frac{\nu }{\mu }-2$ for $m_E(t)$ (when $\frac{\nu }{\mu }>2$) is significant, since this is 1 less than the value $\mu +\frac{\nu }{\mu }-1$ which is the mean “excess degree” of the network (for a node reach by following a random edge, the excess degree is the number of other edges that node has Newman 2018). In other words, non-initial infected nodes always have on average one less susceptible neighbor than they could at their maximum.

We now examine the behavior of $m_E(t)$ as $t\rightarrow \infty$, seeing that it settles at a positive value if and only if $\psi ''(\theta (\infty ))<\mu$ (which we note is also the same condition that governs the final behavior of *m*(*t*)), otherwise $m_E(t)$ vanishes to 0.

### Theorem 9

45 $$\begin{aligned} \lim \limits _{t\rightarrow \infty }m_E(t)=\max \left\{ \theta (\infty )\left( \frac{\psi ''(\theta (\infty ))}{\mu }-1\right) ,0\right\} <\frac{\gamma }{\beta }. \end{aligned}$$

### Proof

Since the rate $\frac{J}{I}+\beta -\frac{\psi ''(\theta )}{\psi '(\theta )}\dot{\theta }$ is always at least $\beta >0$, $m_E(t)$ will always reach the final value of its moving target $\frac{J/I}{J/I+\beta -\frac{\psi ''(\theta )}{\psi '(\theta )}\dot{\theta }}\frac{\psi ''(\theta )\theta }{\psi '(1)}$ in the limit as $t\rightarrow \infty$. Noting that $\lim \limits _{t\rightarrow \infty }\frac{J(t)}{I(t)}=\max \left\{ \beta \left( \frac{\psi ''(\theta (\infty ))}{\psi '(1)}-1\right) ,0\right\}$ (Lemma 2) and $\lim \limits _{t\rightarrow \infty }\dot{\theta }(t)=0$, and then simplifying the moving target accordingly, gives the desired result.

To see that $\theta (\infty )\left( \frac{\psi ''(\theta (\infty ))}{\mu }-1\right) <\frac{\gamma }{\beta }$, realize that $\psi ''(\theta (\infty ))<\frac{\psi '(1)-\psi '(\theta (\infty ))}{1-\theta (\infty )}$ by the convexity of $\psi '$ so that

46 $$\begin{aligned} \frac{\beta }{\beta +\gamma }\frac{\psi ''(\theta (\infty ))}{\psi '(1)}<\frac{\beta }{\beta +\gamma }\frac{\psi '(1)-\psi '(\theta (\infty ))}{\psi '(1)(1-\theta (\infty ))}=1, \end{aligned}$$

the last step of which follows from Eq. (7). The desired inequality than immediately follows. $\square$

This result is demonstrated in Fig. 5, where only the negative binomial network with high dispersion satisfies $\psi ''(\theta (\infty ))>\mu$ (as shown by supplementary Fig. S4), and this is also the only network for which $m_E(t)$ is seen to stay above 0 as $t\rightarrow \infty$.

## The Secondary Case Distribution *Z*(*t*)

Lastly, we introduce the *secondary case distribution* *Z*(*t*), which we define to be the distribution of secondary cases a node infected at time *t* will produce over the course of its infectious period. *Z*(*t*) most directly captures superspreading: what is really important is not that some people are connected more than others, or that they expose susceptible individuals more than others, but rather that they *infect* more than others. This is often how superspreading is defined in the literature in more simple branching process models, as the distribution of secondary cases each infection causes (Lloyd-Smith et al. 2005). However, such models typically ignore time-varying susceptibility and finite population sizes, thus making our formulation of the secondary case distribution on network epidemics novel. Although *Z*(*t*) is in some sense the most natural definition of superspreading we consider, it is also the least analytically tractable, and we are unable to derive a simple differential equation formulation for even its mean $m_Z(t)$.

We also note that *Z*(*t*) is defined based on newly infected nodes at time *t* (thus excluding initially infected nodes), while the other distributions *X*(*t*) and *E*(*t*) are defined for all nodes currently infected at time *t* (though in the supplement we also define and explore analog distributions to *X*(*t*) and *E*(*t*) which are defined for newly infected nodes at time *t* instead). This difference is necessary for defining the secondary case distribution since it is concerned with all future transmissions an infected node causes. It thus makes sense to start tracking these transmissions as soon as the node becomes infected; the infected degree distribution and the effective degree distribution, however, are only concerned with the current state of an infected node and its neighbors at time *t*, which is important to track at different points of an infected node’s infectious period. As we will show, this distinction has important consequences for the behavior of *Z*(*t*).

**Fig. 6:**
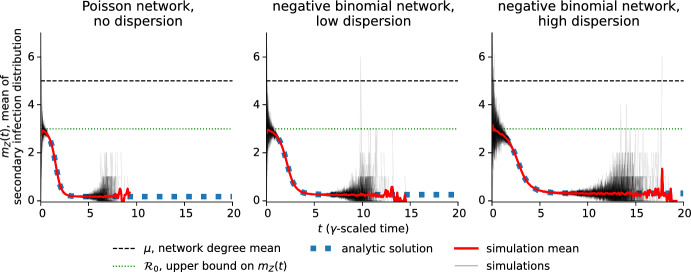
Trajectories of the secondary case distribution’s mean $m_Z(t)$ for three different network degree distributions. Red curves show the mean of 200 simulations (whose individual trajectories are also plotted by faint gray curves) while the blue dotted curves show the analytic solution from Eq. (49). Black dashed lines show the mean $\mu$ of the network degree distribution and green dotted lines show the basic reproduction number $\mathcal {R}_0$ (color figure online)

In order to derive its mass function $p_z(t)$, we first define the quantity $\zeta _t^k(s)$, which is the probability that a node *x* which becomes infected at time $t>0$ and recovers at time *s* infects an arbitrary degree-*k* neighbor *y* (other than the neighbor which infected *x*) by time *s*. This can be calculated using Bayes’ law as follows, noting that *y* cannot have infected by *x* by time *t* and *x* cannot have infected *y* by time *t*:

$$\begin{aligned}&\zeta _t^k(s)=\mathbb {P}\big (y\text { susceptible at time }t\mid y\text { never infected }x\text { by time }t\big )\ \times \\&\quad \int _t^s\mathbb {P}\big (x\text { infects }y\text { in time }(\tau ,\tau +d\tau )\mid y\text { not infected by any other neighbor by time }\tau \big )\ \times \\&\hspace{9.5mm}\mathbb {P}\big (y\text { not infected by any other neighbor by time }\tau \mid y\text { susceptible at time t}\big )\\&\phantom {\zeta _t^k(s)}=\frac{\theta (t)^{k-1}}{\theta (t)}\int _t^s\beta e^{-\beta (\tau -t)}\frac{\theta (\tau )^{k-1}}{\theta (t)^{k-1}}\ d\tau \\&\phantom {\zeta _t^k(s)}=\frac{1}{\theta (t)}\int _t^s\beta e^{-\beta (\tau -t)}\theta (\tau )^{k-1}\ d\tau . \end{aligned}$$

From this, we then define $\zeta _t(s)$ to be the same quantity but with *y* an arbitrary neighbor of *x* of any degree, so that its degree *k* is now distributed according to the neighbor degree distribution $\frac{P(k)k}{\mu }$. Then

47 $$\begin{aligned} \zeta _t(s)=\sum _{k=0}^\infty \frac{P(k)k}{\mu }\zeta _t^k(s)=\frac{1}{\theta (t)}\int _t^s\beta e^{-\beta (\tau -t)}\frac{\psi '(\theta (\tau ))}{\psi '(1)}\ d\tau . \end{aligned}$$

Now consider a node *x* newly infected at time *t* and with degree *j*. One of its *j* neighbors must be the one that transmitted infection to it, so *x* can transmit infection to a maximum of $j-1$ of its neighbors. And for each of these $j-1$ neighbors there is probability $\zeta _t(s)$ that *x* will infect each neighbor, given that *x* recovers at time *s*. Since the probability of *x* recovering at a time in the range $(s,s+ds)$ is $\gamma e^{-\gamma (s-t)}ds$, then the probability that *x* infects exactly *k* of its neighbors during its infectious period is $$\int _t^\infty \gamma e^{-\gamma (s-t)}\left( {\begin{array}{c}j-1\\ k\end{array}}\right) \zeta _t(s)^k(1-\zeta _t(s))^{j-1-k}ds$$.

Now this *x* newly infected at time *t* will have degree *j* with probability $\frac{J_j(t)}{J(t)}=\frac{j\theta (t)^{j-1}}{\psi '(\theta (t))}$, since *J*(*t*) is the number of new infections at time *t* while $J_j$ is the number of those which have degree *j*. Finally, this gives the probability a node newly infected at time *t* will infect exactly *k* neighbors during its infectious period as

48 $$\begin{aligned} p_{Z,k}(t)=\sum _{j=k+1}^\infty \frac{j\theta (t)^{j-1}}{\psi '(\theta (t))}\int _t^\infty \gamma e^{-\gamma (s-t)}\left( {\begin{array}{c}j-1\\ k\end{array}}\right) \zeta _t(s)^k(1-\zeta _t(s))^{j-1-k}ds. \end{aligned}$$

With the mass function derived for the secondary case distribution now derived, we now show that its mean $m_Z(t)$ can be expressed more nicely, but still falls short of a simple differential equation formulation due to the presence of integrals from *t* to infinity.

### Theorem 10

The mean $m_Z(t)$ of the secondary case distribution *Z*(*t*) satisfies

49 $$\begin{aligned} m_Z(t)=\frac{\psi ''(\theta (t))}{\psi '(\theta (t))}\int _t^\infty \beta e^{-(\beta +\gamma )(\tau -t)}\frac{\psi '(\theta (\tau ))}{\psi '(1)}\ d\tau , \end{aligned}$$

and if the function $\psi '$ is log-convex, then the initial value of $m_Z$ satisfies

50 $$\begin{aligned} m_Z(0)<\mathcal {R}_0. \end{aligned}$$

### Proof

The equation for the mean follows by plugging Eq. (48) into $m_Z(t)=\sum _{k=0}^\infty p_{E,k}(t)$, with the sums of the binomial terms simplifying to the expectations of binomial random variables. Now the condition that $\psi '$ is log-convex implies $\frac{\psi ''(\theta (t))}{\psi '(\theta (t))}<\frac{\psi ''(1)}{\psi '(1)}$ for all *t*, so that $m_Z(0)<\frac{\psi ''(1)}{\psi '(1)}\int _0^\infty \beta e^{-(\beta +\gamma )\tau }\ d\tau =\frac{\beta }{\beta +\gamma }\frac{\psi ''(1)}{\psi '(1)}=\mathcal {R}_0$. $\square$

We note that this log-convexity condition for $\psi '$ will only ever be met by network degree distributions with infinite support, but most distributions commonly used to model contact networks that have defined variance (precluding scale-free networks) have this log-convexity property for the derivative of their probability-generating function, including Poisson, negative binomial, and geometric distributions.

The higher moments of *Z*(*t*) are even less tractable, but we provide a formula for the second moment $m_{Z,2}(t)$ in the supplement, from which the variance can be calculated as $v_Z(t)=m_{Z,2}(t)-m_Z(t)^2$. We show the trajectories of $m_Z(t)$ in Fig. 6 and the trajectories of $v_Z(t)$ in y Fig. S3.

The peak behavior of $m_Z(t)$ is not as interesting as with *m*(*t*) or $m_E(t)$. Again, assuming log-convexity of $\psi '$, it is easy to see from Eq. (49) that $m_Z(t)$ will always be decreasing:

### Theorem 11

Assuming $\psi '$ is log-convex, $m_Z(t)$ will always be decreasing for all *t*.

### Proof

This follows from the facts that $\frac{\psi ''(\theta (t))}{\psi '(\theta (t))}$ is decreasing, by the log-convexity of $\psi '$, and that $\psi '(\theta (\tau ))$ is decreasing with $\tau$. $\square$

This result makes intuitive sense if $m_Z(t)$ is compared to the notion of the “effective reproduction number” $\mathcal {R}(t)$, which is typically defined to be the average number of secondary cases produced by individuals infected at time *t* over the course of their infectious period if population susceptibility were to stay constant from time *t* onward. This last condition of susceptibility staying constant is the only difference conceptually between $m_Z(t)$ and $\mathcal {R}(t)$, and this should ensure that $m_Z(t)<\mathcal {R}(t)$ for all *t*. Thus, it makes sense that $m_Z(t)$ declines for all *t* and $m_Z(0)<\mathcal {R}_0$, since $\mathcal {R}(t)$ generally declines for all *t* in epidemics without demographics or waning immunity and $\mathcal {R}(0)\approx \mathcal {R}_0$ by definition.

As mentioned before, *Z*(*t*) is fundamentally different from *X*(*t*) and *E*(*t*) in its focus on newly infected nodes at time *t* rather than at all currently infected nodes at time *t*. This distinction is responsible for the difference in peak behavior between $m_Z(t)$ and the means *m*(*t*) and $m_E(t)$ of the other distributions. At the start, most of the nodes *X*(*t*) and *E*(*t*) consider are initial infections, whose average degree ($\mu$) is less than the average degree of the newly infected nodes near the start ($\mu +\frac{\nu }{\mu }$). However, *Z*(*t*) only considers these newly infected nodes, and its calculation never considers the initial infections.

Finally, we provide the behavior of $m_Z(t)$ as $t\rightarrow \infty$, which always converges to a positive final value that is less than 1:

### Theorem 12

51 $$\begin{aligned} \lim _{t\rightarrow \infty }m_Z(t)=\frac{\beta }{\beta +\gamma }\frac{\psi ''(\theta (\infty ))}{\mu }<1. \end{aligned}$$

### Proof

In the limit as $t\rightarrow \infty$, we substitute $\theta (\infty )$ in for $\theta (t)$ in Eq. (49), which gives $\lim \limits _{t\rightarrow \infty }m_Z(t)=\lim \limits _{t\rightarrow \infty }\frac{\psi ''(\theta (\infty ))}{\psi '(\theta (\infty ))}\frac{\psi '(\theta (\infty ))}{\psi '(1)}\int _t^\infty \beta e^{-(\beta +\gamma )(\tau -t)}d\tau$ and thus the desired result. And we have previously shown that this quantity is less than 1 in Eq. (46). $\square$

This fact also aligns with the closely related effective reproduction number $\mathcal {R}$, which also always reaches a final value less than 1 at the end of a non-endemic epidemic.

**Fig. 7:**
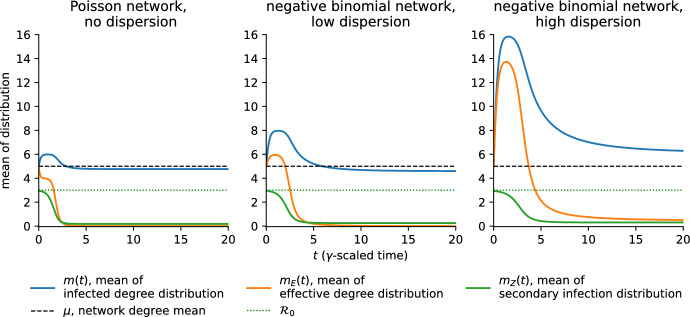
Analytic trajectories of the three superspreading metrics—the mean *m*(*t*) of the infected degree distribution, the mean $m_E(t)$ of the effective degree distribution, and the mean $m_Z(t)$ of the secondary case distribution—we define in Eq. (20), Eq. (39), and Eq. (49), respectively, and for three different network degree distributions. Black dashed lines show the mean $\mu$ of the network degree distribution and green dotted lines show the basic reproduction number $\mathcal {R}_0$ (color figure online)

## Discussion

While it has been known in both the theoretical and empirical literature that the potential for superspreading in an epidemic declines over time, we provide the first rigorous mathematical demonstration of this fact in a model of a network epidemic, specifically utilizing the edge-based framework of Miller (2011); Miller et al. (2012). Depending on how the “potential for superspreading” is defined, however, can yield different qualitative results, as we show. We define three reasonable metrics for tracking superspreading over time: the mean *m*(*t*) of the infected degree distribution, the mean $m_E(t)$ of the effective degree distribution, and the mean $m_Z(t)$ of the secondary case distribution (shown all together in Fig. 7). These metrics measure the expected number of contacts involving infectious individuals, expected exposures involving susceptible and infectious individuals, and expected transmissions; in order of increasing accordance with the definition of superspreading but also in order of decreasing analytic tractability. Yet, we are still able to show that these three metrics differ in their behaviors in crucial ways. Notably, while all three metrics do decline eventually, signaling a decreasing role of superspreading, *m*(*t*) and $m_E(t)$ can have an initial increase and noticeable peaks. We show that the peak times of these metrics all occur in less than half the time it takes for population-level prevalence to peak in an epidemic, suggesting that the role of superspreading declines well before an epidemic reaches its most severe.

This result implies that the efficacy of contact-based control strategies (Nielsen et al. 2021; Sneppen et al. 2021; Kain et al. 2021; Boudreau et al. 2023) to minimize the role of superspreading are highly dependent on the timing of the intervention. Specifically, trying to control superspreading events via contact-based interventions may have little effect once the potential for superspreading has died down. And, as we have shown, this happens rather early compared to the overall dynamics of the epidemic, for all metrics we consider (Fig. 3). After infections have reached a considerable level in the population, it may be more effective to switch to other strategies that aim to uniformly curb transmission among all individuals rather than trying to target those with more contacts.

Our results also have important implications for the accuracy of methods that aim to measure and quantify superspreading. Most commonly, dispersion is measured by estimating the distribution of secondary cases caused by infected individuals: This can be done directly with transmission trees inferred from contact tracing or sequencing data (Adam et al. 2020), or by simulating epidemic models to reproduce observed incidence (Riou and Althaus 2020), which can give similar results (Hébert-Dufresne 2021). These methods correspond to estimating the secondary case distribution, *Z*(*t*). On the other hand, some methods instead attempt to measure dispersion by estimating the distribution of the total number of contacts infected individuals have (some of which may not lead to transmissions), through the use of mobility data (Lau et al. 2020). This corresponds more to estimating the infected degree distribution *X*(*t*). However, as we have shown, the temporal properties and magnitudes of *Z*(*t*) and *X*(*t*) can differ substantially. Thus, parameter inference may depend greatly on the distribution underlying the method, whether it is concerned with infected individuals’ secondary transmissions or their total contacts.

Furthermore, we show that these distributions can change drastically over the course of an epidemic, especially in the early stages, as population susceptibility shifts from higher degree to lower degree nodes. Thus, regardless of the method used to infer dispersion, the data available is likely to involve a significant time period over which the importance of superspreading will have changed. Using fine-grained temporal data (on incidence or contact tracing) might limit the statistical power of the method, but coarse-graining the data involves averaging over very different superspreading patterns. Consequently, it is common to separate an epidemic in multiple periods, sometimes simply in half with two phases of rising or decreasing incidence (Ko et al. 2022). In our simple framework, we show that the second half of the timeframe (and therefore more than half of available case data) will show significantly lower superspreading than what drove the early epidemic dynamics. Future work could use our framework to redefine epidemic phases based on the varying importance of superspreading over time. In doing so, we could redesign inference methods, forecasting models, and intervention strategies to better adapt to the time-varying statistical patterns of epidemics.

Our results may be altered by static and dynamic modifications to the network structure. For example, generalizing away from the simple configuration model while keeping the network structure static, one may examine how clustering may affect the intensity and timing of superspreading. Our methods to analyze superspreading through the degree distribution of infected nodes could easily be extended to networks with clustering through the use of previous work on incorporating clustering into the edge-based epidemic modeling framework (Volz et al. 2011). We expect that highly clustered networks would dampen and delay the impact of superspreading, as the neighbors of a high degree node would be more likely to be connected to each other and infect each other before the high degree node can. Furthermore, networks may change their structure throughout the course of an epidemic, either through random shuffling (Miller et al. 2012; Lamata-Otín et al. 2026) or adaptive behavior (Gross et al. 2006; Althouse and Hébert-Dufresne 2014; Scarpino et al. 2016). The impact of network restructuring on superspreading intensity and timing would likely depend on how the restructuring is modeled: For example, infected nodes restricting their contacts to simulate self-isolation may reduce superspreading potential, while nodes moving contacts from infected neighbors to new susceptible neighbors could create high degree susceptible nodes and increased superspreading potential late in an epidemic (Gross et al. 2006; Scarpino et al. 2016). Such modifications to the network structure, both static and dynamic, would be promising candidates for extending our superspreading analysis to more general contexts.

## Data Availability

All code to run the analyses and produce the figures in this paper can be found at https://github.com/freedmanari/infected_degrees.

## References

[CR1] Adam DC et al (2020) Clustering and superspreading potential of SARS-CoV-2 infections in hong kong. Nat Med 26(11):1714–1719 32943787 10.1038/s41591-020-1092-0

[CR2] Allard A, Moore C, Scarpino SV, Althouse BM, Hébert-Dufresne L (2023) The Role of Directionality, Heterogeneity, and Correlations in Epidemic Risk and Spread. SIAM Rev 65(2):471–492

[CR3] Althouse BM et al (2020) Superspreading events in the transmission dynamics of SARS-CoV-2: Opportunities for interventions and control. PLoS Biol 18(11):e3000897 33180773 10.1371/journal.pbio.3000897 PMC7685463

[CR4] Althouse BM, Hébert-Dufresne L (2014) Epidemic cycles driven by host behaviour. J R Soc Interface 11(99):20140575 25100316 10.1098/rsif.2014.0575 PMC4235258

[CR5] Andersson H (1997) Epidemics in a population with social structures. Math Biosci 140(2):79–84 9046769 10.1016/s0025-5564(96)00129-0

[CR6] Balcan D et al (2009) Multiscale mobility networks and the spatial spreading of infectious diseases. Proc Natl Acad Sci 106(51):21484–21489 20018697 10.1073/pnas.0906910106 PMC2793313

[CR7] Ball F, Neal P (2008) Network epidemic models with two levels of mixing. Math Biosci 212(1):69–87 18280521 10.1016/j.mbs.2008.01.001

[CR8] Barrat A, Barthélemy M, Vespignani A (2013) Dynamical Processes on Complex Networks. (Cambridge University, Cambridge, UK) Vol. 37. Publisher: Routledge _eprint: 10.1080/0022250X.2012.728886

[CR9] Barthélemy M, Barrat A, Pastor-Satorras R, Vespignani A (2005) Dynamical patterns of epidemic outbreaks in complex heterogeneous networks. J Theor Biol 235(2):275–288 15862595 10.1016/j.jtbi.2005.01.011

[CR10] Bauch C (2002) A versatile ODE approximation to a network model for the spread of sexually transmitted diseases. J Math Biol 45(5):375–395 12424529 10.1007/s002850200153

[CR11] Boudreau MC, Allen AJ, Roberts NJ, Allard A, Hébert-Dufresne L (2023) Temporal and probabilistic comparisons of epidemic interventions. Bull Math Biol 85(12):118 37857996 10.1007/s11538-023-01220-w PMC11216031

[CR12] Brainard J, Jones NR, Harrison FCD, Hammer CC, Lake IR (2023) Super-spreaders of novel coronaviruses that cause SARS, MERS and COVID-19: a systematic review. Ann Epidemiol 82:66-76.e6 37001627 10.1016/j.annepidem.2023.03.009 PMC10208417

[CR13] Britton T, Ball F, Trapman P (2020) A mathematical model reveals the influence of population heterogeneity on herd immunity to SARS-CoV-2. Science 369(6505):846–849 32576668 10.1126/science.abc6810 PMC7331793

[CR14] Diekmann O, Heesterbeek H, Britton T (2012) Mathematical Tools for Understanding Infectious Disease Dynamics. Google-Books-ID: XbntAQAAQBAJ. Princeton University Press,

[CR15] Eames KTD, Keeling MJ (2002) Modeling dynamic and network heterogeneities in the spread of sexually transmitted diseases. Proc Natl Acad Sci 99(20):13330–13335 12271127 10.1073/pnas.202244299 PMC130633

[CR16] Endo A, Abbott S, Kucharski AJ, Funk S (2020) Estimating the overdispersion in COVID-19 transmission using outbreak sizes outside China. Wellcome Open Research, 10.12688/wellcomeopenres.15842.1 PMC7338915 32685698

[CR17] Eubank S et al (2004) Modelling disease outbreaks in realistic urban social networks. Nature 429(6988):180–184 15141212 10.1038/nature02541

[CR18] Gleeson JP (2013) Binary-State Dynamics on Complex Networks: Pair Approximation and Beyond. Phys Rev X 3(2):021004

[CR19] Gomes MGM et al (2022) Individual variation in susceptibility or exposure to SARS-CoV-2 lowers the herd immunity threshold. J Theor Biol 540:111063 35189135 10.1016/j.jtbi.2022.111063 PMC8855661

[CR20] Goyal A, Reeves DB, Cardozo-Ojeda EF, Schiffer JT, Mayer BT (2021) Viral load and contact heterogeneity predict SARS-CoV-2 transmission and super-spreading events. eLife 10:e63537 10.7554/eLife.63537 PMC7929560 33620317

[CR21] Gross T, D’Lima CJD, Blasius B (2006) Epidemic Dynamics on an Adaptive Network. Phys Rev Lett 96(20):208701 16803215 10.1103/PhysRevLett.96.208701

[CR22] Großmann G, Backenköhler M, Wolf V (2021) Heterogeneity matters: Contact structure and individual variation shape epidemic dynamics. PLoS ONE 16(7):e0250050 34283842 10.1371/journal.pone.0250050 PMC8291658

[CR23] Guo Z et al (2023) A statistical framework for tracking the time-varying superspreading potential of COVID-19 epidemic. Epidemics 42:100670 36709540 10.1016/j.epidem.2023.100670 PMC9872564

[CR24] Hébert-Dufresne L (2021) The network epidemiology of an Ebola epidemic.arXiv:2111.08686 arXiv preprint

[CR25] Hébert-Dufresne L, Noël PA, Marceau V, Allard A, Dubé LJ (2010) Propagation dynamics on networks featuring complex topologies. Phys Rev E 82(3):036115 10.1103/PhysRevE.82.036115 21230147

[CR26] Heesterbeek H (2005) 5 - THE LAW OF MASS-ACTION IN EPIDEMIOLOGY: A HISTORICAL PERSPECTIVE in Ecological Paradigms Lost, Theoretical Ecology Series, eds. Cuddington K, Beisner BE. (Academic Press, Burlington), pp. 81–105

[CR27] Kain MP, Childs ML, Becker AD, Mordecai EA (2021) Chopping the tail: How preventing superspreading can help to maintain COVID-19 control. Epidemics 34:100430 33360871 10.1016/j.epidem.2020.100430 PMC7833509

[CR28] Karrer B, Newman M (2010) Message passing approach for general epidemic models. Phys Rev E 82(1):10.1103/PhysRevE.82.016101 20866683

[CR29] Kermack WO, McKendrick AG (1927) A contribution to the mathematical theory of epidemics. Proceedings of the Royal Society of London. Series A, Containing Papers of a Mathematical and Physical Character 115(772):700–721

[CR30] Kiss IZ, Green DM, Kao RR (2005) Infectious disease control using contact tracing in random and scale-free networks. J R Soc Interface 3(6):55–62 10.1098/rsif.2005.0079 PMC1618487 16849217

[CR31] Kiss IZ, Kenah E, Rempała GA (2023) Necessary and sufficient conditions for exact closures of epidemic equations on configuration model networks. J Math Biol 87(2):36 37532967 10.1007/s00285-023-01967-9 PMC10397147

[CR32] Klovdahl AS (1985) Social networks and the spread of infectious diseases: The AIDS example. Social Science & Medicine 21(11):1203–1216 3006260 10.1016/0277-9536(85)90269-2

[CR33] Ko YK et al (2022) Secondary transmission of SARS-CoV-2 during the first two waves in Japan: Demographic characteristics and overdispersion. Int J Infect Dis 116:365–373 35066162 10.1016/j.ijid.2022.01.036 PMC8772065

[CR34] Lamata-Otín S, et al. (2026) Group dynamics shape contagion onsets and multistable active phases under collective reinforcement. Version Number: 1

[CR35] Lau MSY et al (2020) Characterizing superspreading events and age-specific infectiousness of SARS-CoV-2 transmission in Georgia, USA. Proc Natl Acad Sci 117(36):22430–22435 32820074 10.1073/pnas.2011802117 PMC7486752

[CR36] Leventhal GE, Hill AL, Nowak MA, Bonhoeffer S (2015) Evolution and emergence of infectious diseases in theoretical and real-world networks. Nature Communications 6(1):6101. Number: 1 10.1038/ncomms7101 PMC4335509 25592476

[CR37] Lindquist J, Ma J, van den Driessche P, Willeboordse FH (2011) Effective degree network disease models. J Math Biol 62(2):143–164 20179932 10.1007/s00285-010-0331-2

[CR38] Lloyd-Smith JO, Schreiber SJ, Kopp PE, Getz WM (2005) Superspreading and the effect of individual variation on disease emergence. Nature 438(7066):355–359 16292310 10.1038/nature04153 PMC7094981

[CR39] Marceau V, Noël PA, Hébert-Dufresne L, Allard A, Dubé LJ (2010) Adaptive networks: Coevolution of disease and topology. Phys Rev E 82(3):036116 10.1103/PhysRevE.82.036116 21230148

[CR40] May RM, Lloyd AL (2001) Infection dynamics on scale-free networks. Phys Rev E 64(6):066112 10.1103/PhysRevE.64.066112 11736241

[CR41] Miller JC (2011) A note on a paper by Erik Volz: SIR dynamics in random networks. J Math Biol 62(3):349–358 20309549 10.1007/s00285-010-0337-9

[CR42] Miller D et al (2020) Full genome viral sequences inform patterns of SARS-CoV-2 spread into and within Israel. Nat Commun 11(1):5518 33139704 10.1038/s41467-020-19248-0 PMC7606475

[CR43] Miller JC, Ting T (2019) EoN (Epidemics on Networks): a fast, flexible Python package for simulation, analytic approximation, and analysis of epidemics on networks. Journal of Open Source Software 4(44):1731

[CR44] Miller JC, Slim AC, Volz EM (2012) Edge-based compartmental modelling for infectious disease spread. J R Soc Interface 9(70):890–906 21976638 10.1098/rsif.2011.0403 PMC3306633

[CR45] Miyama T, Sm J, Nishiura H (2022) Decrease in overdispersed secondary transmission of COVID-19 over time in Japan. Epidemiology & Infection 150:e197 36377373 10.1017/S0950268822001789 PMC9744460

[CR46] Newman MEJ (2002) Spread of epidemic disease on networks. Phys Rev E 66(1):016128 10.1103/PhysRevE.66.016128 12241447

[CR47] Newman M (2018). Oxford University Press,(Networks. 2nd edition)

[CR48] Nielsen BF et al (2023) Host heterogeneity and epistasis explain punctuated evolution of SARS-CoV-2. PLoS Comput Biol 19(2):e1010896 36791146 10.1371/journal.pcbi.1010896 PMC9974118

[CR49] Nielsen BF, Simonsen L, Sneppen K (2021) COVID-19 Superspreading Suggests Mitigation by Social Network Modulation. Phys Rev Lett 126(11):118301 33798363 10.1103/PhysRevLett.126.118301

[CR50] Noël PA, Davoudi B, Brunham RC, Dubé LJ, Pourbohloul B (2009) Time evolution of epidemic disease on finite and infinite networks. Phys Rev E 79(2):026101 10.1103/PhysRevE.79.026101 19391800

[CR51] Oz Y, Rubinstein I (2021) Safra M (2021) Heterogeneity and superspreading effect on herd immunity. J Stat Mech: Theory Exp 3:033405

[CR52] Pastor-Satorras R, Vespignani A (2002) Epidemic dynamics in finite size scale-free networks. Phys Rev E 65(3):035108 10.1103/PhysRevE.65.035108 11909143

[CR53] Riou J, Althaus CL (2020) Pattern of early human-to-human transmission of Wuhan 2019 novel coronavirus (2019-nCoV), December 2019 to January 2020. Eurosurveillance 25(4):2000058 32019669 10.2807/1560-7917.ES.2020.25.4.2000058 PMC7001239

[CR54] Scarpino SV, Allard A, Hébert-Dufresne L (2016) The effect of a prudent adaptive behaviour on disease transmission. Nat Phys 12(11):1042–1046

[CR55] Sherborne N, Miller JC, Blyuss KB, Kiss IZ (2018) Mean-field models for non-Markovian epidemics on networks. J Math Biol 76(3):755–778 28685365 10.1007/s00285-017-1155-0 PMC5772140

[CR56] Sneppen K, Nielsen BF, Taylor RJ, Simonsen L (2021) Overdispersion in COVID-19 increases the effectiveness of limiting nonrepetitive contacts for transmission control. Proc Natl Acad Sci 118(14):e2016623118 33741734 10.1073/pnas.2016623118 PMC8040586

[CR57] Volz E (2008) SIR dynamics in random networks with heterogeneous connectivity. J Math Biol 56(3):293–310 17668212 10.1007/s00285-007-0116-4 PMC7080148

[CR58] Volz EM, Miller JC, Galvani A, Ancel Meyers L (2011) Effects of Heterogeneous and Clustered Contact Patterns on Infectious Disease Dynamics. PLoS Comput Biol 7(6):e1002042 21673864 10.1371/journal.pcbi.1002042 PMC3107246

[CR59] Watts DJ, Strogatz SH (1998) Collective dynamics of ‘small-world’ networks. Nature 393(6684):440–442 9623998 10.1038/30918

